# Crystal structure of {*N*-[(6-bromo­pyridin-2-yl)(phen­yl)methyl­idene]-2,6-di­methyl­aniline-κ^2^ *N*,*N*′}di­chlorido­zinc di­chloro­methane hemisolvate

**DOI:** 10.1107/S2056989017007812

**Published:** 2017-06-02

**Authors:** Bradley M. Wile

**Affiliations:** a Donald J. Bettinger Department of Chemistry and Biochemistry, Ohio Northern University, 525 S. Main Street, Ada, OH 45810, USA

**Keywords:** crystal structure, imino­pyridine, redox-active ligand, coordination compound, zinc(II), pyridyl halide

## Abstract

The solvated title compound, [ZnCl_2_(C_20_H_17_BrN_2_)]·0.5CH_2_Cl_2_, comprises a bidentate imino­pyridine ligand and two Cl atoms bound to a zinc^2+^ cation in a distorted tetra­hedral arrangement. The chelate bond lengths are consistent with localized C=N double bonds and a C—C single bond, as expected for an unreduced ligand bound to a closed-shell transition metal cation. Apart from weak nonclassical C—H⋯Cl hydrogen bonds between the complex mol­ecules and the disordered solvent mol­ecules (occupancy = 0.5), no further significant inter­molecular inter­actions are observed.

## Abstract 2

The title compound consists of a bidentate α-imino­pyridine ligand and two Cl atoms bound to a zinc(II) cation. Chelate bond lengths are consistent with an unreduced ligand bound to the *d* ^10^ zinc(II) cation.

## Chemical context   

Redox-active ligands bearing an α-imino­pyridine core have received much attention in the literature (Bianchini *et al.*, 2007 ▸; Lu *et al.*, 2008 ▸). While most α-iminopyridine ligands reported to date feature a methyl imine ‘backbone’, a small number of variants featuring more electron-withdrawing phenyl backbones have been reported (Archer *et al.*, 2006 ▸; Tondreau *et al.*, 2013 ▸; Yang *et al.*, 2010 ▸). Single-crystal X-ray diffraction studies have been a critical component in the elucidation of the electronic structure of base metal complexes featuring these redox-active ligands (Bart *et al.*, 2006 ▸; Lu *et al.*, 2008 ▸; Tondreau *et al.*, 2013 ▸). A comparison of the N_imine_—C_imine_, C_imine_—C_*ipso*_, and C_*ipso*_—N_pyridine_ bond lengths for reduced and unreduced ligands as free bases or closed-shell complexes facilitate conclusions about redox non-innocence for such ligand sets. To this end, the preparation of the titular zinc(II) complex featuring the unreduced ligand was undertaken. Inclusion of a bromine functionality in the remaining *ortho* position of the pyridine ring allows for the introduction of an additional donor arm that differs from the imine fragment (Zhang & Lu, 2016 ▸; He *et al.*, 2016 ▸).

## Structural commentary   

**Figure 1:**
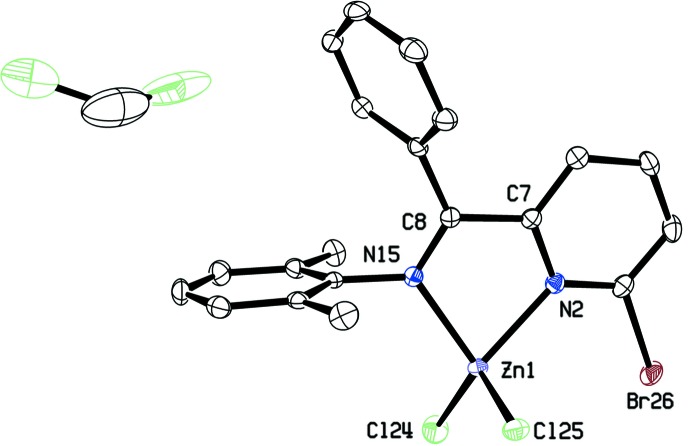
The mol­ecular stucture of **3-ZnCl_2_**, with displacement ellipsoids shown at the 30% probability level and a partial numbering scheme. H atoms have been omitted for clarity. Cocrystallized CH_2_Cl_2_ solvent (disordered) is present in the ratio **3-ZnCl_2_·0.5CH_2_Cl_2_**.

The mol­ecular structure of the titular compound is shown in Fig. 1 ▸. In this complex, the Zn^2+^ cation adopts a distorted tetra­hedral arrangement (Table 1 ▸), being surrounded by two Cl atoms and two N atoms. The N atoms comprise the donor atoms for an α-imino­pyridine ligand, forming a five-membered ring when bound to the Zn^2+^ cation (Zn1—N2—C7—C8—N15). The Zn^2+^ cation lies 0.3855 (3) Å above the plane defined by the chelate (N2/C7/C8/N15), in a distorted tetra­hedral arrangement (τ_4_ parameter = 0.8999; Yang *et al.*, 2007 ▸). Distortions to the geometry about the metal cation and the arrangement of the pyridine and phenyl rings [dihedral angle = 66.62 (13)°] may be attributed to the steric pressure exerted by the ligand substituents, and packing constraints within the unit cell.

Bond lengths and angles for the α-imino­pyridine fragment (N2/C7/C8/N15) of the ligand are consistent with the depiction as localized C=N double bonds, and as a C—C single bond. A comparison of the observed bond lengths with the average bond lengths for neutral and doubly-reduced α-imino­pyridine (α-IP; Lu *et al.*, 2008 ▸) and pyridine di­imine (PDI; Bart *et al.*, 2006 ▸) ligands is given in Table 2 ▸.
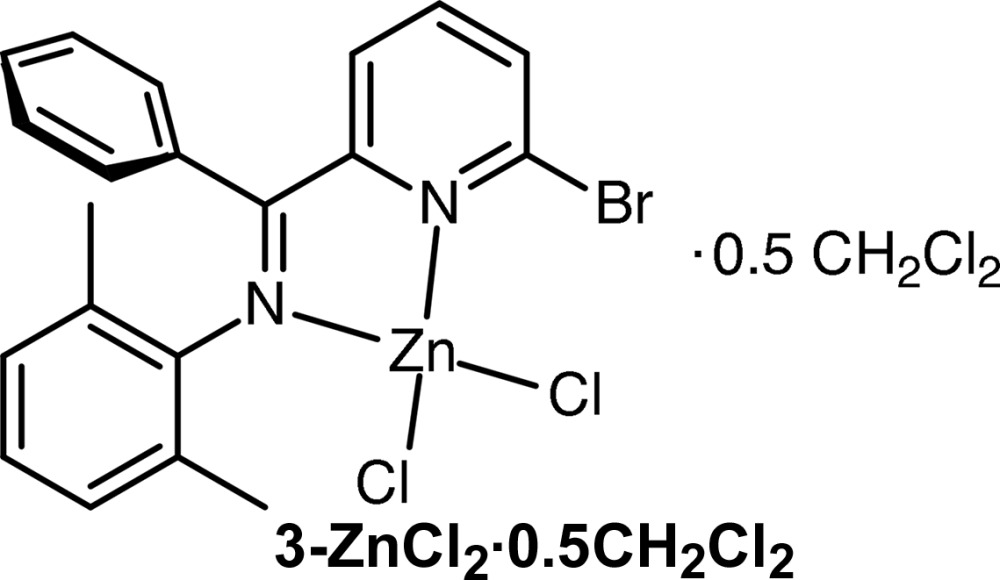


## Supra­molecular features   

**Figure 2:**
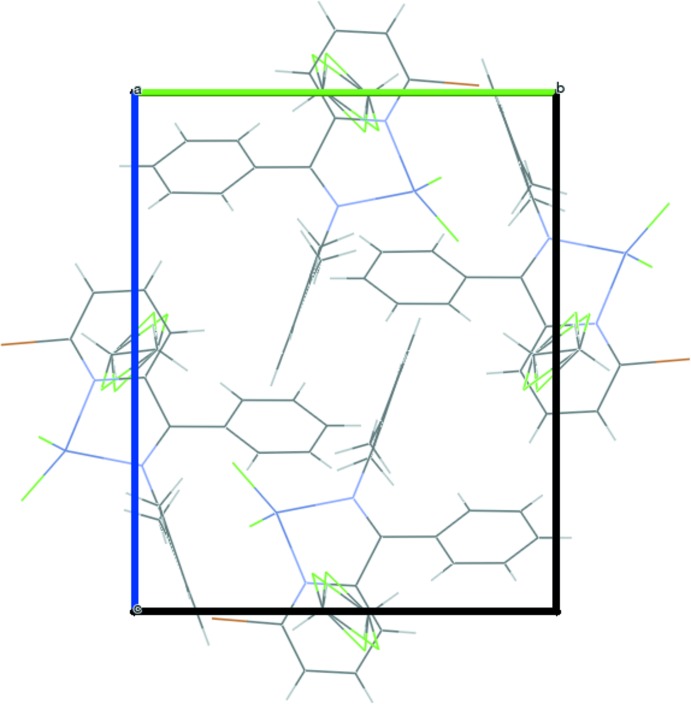
Packing of **3-ZnCl_2_·0.5CH_2_Cl_2_**, viewed along *a*.

One half of a disordered mol­ecule of di­chloro­methane is present in the asymmetric unit, close to a center of inversion. While no hydrogen bonding is observed between the complex mol­ecules in this crystal, several short contacts (less than the sum of the van der Waals radii) are observed between neighbouring mol­ecules. Notably, neither dimerization nor stoichiometric binding of solvent to the metal cation is observed for this complex, in contrast to some base metal complexes of similar ligands (Dai *et al.*, 2016 ▸; Song *et al.*, 2011 ▸). However, a weak C—H⋯Cl inter­action binds the disordered solvent molecule to the complex (Table 3 ▸). Fig. 2 ▸ depicts the packing within the unit cell, as viewed along the *a* axis.

## Synthesis and crystallization   

**Figure 3:**
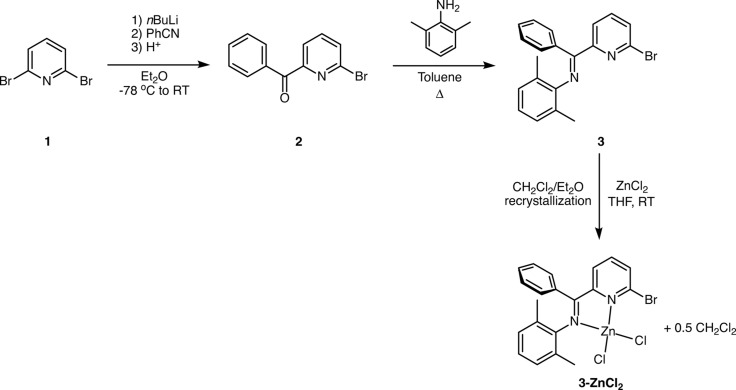
Schematic representation of the preparation of ligand (**3**) and the corresponding zinc(II) complex (**3-ZnCl_2_**).

The titular compound was prepared in good yield using the scheme described in Fig. 3 ▸. Experimental details are described below for each stage of the synthesis.

### Preparation of (6-bromopyridin-2-yl)phenyl ketone, (2)   

Following the method of Kobayashi and co-workers (Ishikawa *et al.*, 2005 ▸), to a solution of 2,6-di­bromo­pyridine (**1**, 10.0 g, 42.2 mmol) in diethyl ether (200 ml) at 195 K, was added *n*-BuLi (29 ml of a 1.6 *M* solution in diethyl ether, 46.4 mmol) dropwise over 5 min. The solution was stirred at 195 K for 1 h, after which benzo­nitrile (4.8 ml, 46.4 mmol) was added dropwise over 5 min. The resultant solution was allowed to warm to room temperature, at which point the yellow solution turned dark red. After 1 h, cold aqueous 3 *M* HCl (250 ml) was added to the solution causing the dark-red solution to turn yellow, and the organic phase was removed. To the aqueous layer, 3 *M* NaOH (250 ml) was added, and the mixture was extracted with diethyl ether (3 × 100 ml). The organic fractions were combined, dried over MgSO_4_, and concentrated under reduced pressure. The product (**2**) was recrystallized from ethanol, yielding a light-yellow crystalline solid (4.35 g, 16.6 mmol, 39%; m.p. 330–333 K).

### Preparation of *N*-[(6-bromo­pyridin-2-yl)(phen­yl)methyl­idene]-2,6-di­methyl­aniline, (3)   

Following the method of Meneghetti *et al.* (1999 ▸), a round-bottomed flask containing **2** (3.00 g, 12.6 mmol), 2,6-di­methyl­aniline (3.15 ml, 25.2 mmol), ∼30 mg of *p*-toluene­sulfonic acid catalyst, and toluene (300 ml) were fitted with a Dean–Stark apparatus, and brought to reflux for 6 d. The mixture was washed with a saturated aqueous solution of NaHCO_3_, dried over MgSO_4_, and concentrated under reduced pressure. The resultant brown (crude) product was purified by column chromatography (silica 50–70 ml) with a 4:1 (*v*/*v*) hexa­nes–ethyl acetate mixture as eluant (*R*_F_ = 0.62) to yield **3** as a bright-yellow solid (2.85 g, 8.4 mmol, 67%; m.p. 361–366 K).

### Preparation of {*N*-[(6-bromo­pyridin-2-yl)(phen­yl)meth­yl­idene]-2,6-di­methyl­aniline-κ^2^ *N*,*N*′}dichloridozinc di­chloro­methane hemisolvate, (3-ZnCl_2_)   

Anhydrous zinc(II) chloride (0.068 g, 0.50 mmol) and **3** (0.237 g, 0.65 mmol) solids were added to a Schlenk flask fitted with a magnetic stirrer bar, and the flask was flushed with argon. Anhydrous tetra­hydro­furan (10 ml) was added to the flask, and the solution was allowed to stir for 16 h. The solvent and other volatiles were removed *in vacuo*, and the residue was rinsed with dry pentane to yield **3-ZnCl_2_** as a yellow solid (0.251 g, 0.50 mmol, >99%). Single crystals suitable for X-ray diffraction were obtained by diffusion of diethyl ether into a saturated solution of **3-ZnCl_2_** in CH_2_Cl_2_. ^1^H NMR (CDCl_3_, 400 MHz; see also supporting information) δ 8.01 (*d*, *J* = 8.0 Hz, 1H, aryl *m*-CH), 7.95 (*t*, *J* = 8.0 Hz, 1H, aryl *p*-CH), 7.60 (*d*, *J* = 7.6 Hz, 1H, aryl *m*-CH), 7.49 (*t*, *J* = 7.2 Hz, 1H, phenyl *p*-CH), 7.39 (*t*, *J* = 7.2 Hz, 2H, phenyl *m*-CH), 7.21 (*d*, *J* = 7.6 Hz, 2H, phenyl *o*-CH), 7.01–6.92 (*m*, 3H, pyridine CH), 5.30 (*s*, 0.5 × 2H, CH_2_Cl_2_), 2.30 (*s*, 6H, CH_3_). ^13^C{^1^H} NMR (CDCl_3_, 100 MHz, see also supporting information): δ 169.2 (C=N), 150.3 (aryl ipso-C), 144.5, 142.4, 142.2 (aryl *p*-CH), 133.9 (aryl *m*-CH), 131.8 (phenyl *p*-CH), 130.4 (phenyl ipso-C), 128.9 (aryl *o*-C), 128.8 (phenyl *m*-CH), 128.6 (pyridine CH), 127.8 (phenyl *o*-CH), 127.0 (aryl *m*-CH and pyridine CH), 19.2 (CH_3_); m.p. 529–537 K. Analysis calculated (%) for C_20.5_H_18_N_2_BrCl_3_Zn: C 45.26, H 3.33, N 5.15; found: C 45.19, H 3.40, N 5.06.

## Refinement   

Crystal data, data collection and structure refinement details are summarized in Table 4 ▸. H atoms were placed in calculated positions, and their positions were initially refined using distance and angle restraints. A disordered mol­ecule of di­chloro­methane was located close to a center of inversion. All atoms from the solvent mol­ecule were refined with a fixed occupancy of 0.5, and SAME and SIMU restraints were employed.

## Supplementary Material

Crystal structure: contains datablock(s) I, global. DOI: 10.1107/S2056989017007812/wm5393sup1.cif

Structure factors: contains datablock(s) I. DOI: 10.1107/S2056989017007812/wm5393Isup2.hkl

NMR spectrum. DOI: 10.1107/S2056989017007812/wm5393sup4.pdf

NMR spectrum. DOI: 10.1107/S2056989017007812/wm5393sup5.pdf

CCDC reference: 1552501

Additional supporting information:  crystallographic information; 3D view; checkCIF report

## Figures and Tables

**Table 1:** Selected geometric parameters (Å, °)

Zn1—N2	2.088 (2)	Zn1—Cl24	2.1761 (7)
Zn1—N15	2.0778 (19)	Zn1—Cl25	2.2281 (7)
			
N2—Zn1—N15	79.01 (8)	N2—Zn1—Cl25	100.92 (6)
N2—Zn1—Cl24	127.60 (6)	N15—Zn1—Cl25	109.18 (6)
N15—Zn1—Cl24	114.61 (6)	Cl24—Zn1—Cl25	118.50 (3)

**Table 2:** Comparison of N_imine_—C_imine_, C_imine_—C_*ipso*_, and C_*ipso*_—N_pyridine_ bond lengths (Å)

Compound	N_imine_—C_imine_	C_imine_—C_*ipso*_	C_*ipso*_—N_pyridine_
α-IP*^*a*^*	1.28	1.47	1.35
α-IP^2−^ ^a^	1.46	1.39	1.40
PDI*^*b*^*	1.271 (17)	1.480 (19)	1.345 (17)
PDI^2−^ ^*b*,*c*^	1.363	1.443	1.332
This work	1.283 (3)	1.500 (4)	1.361 (5)

Notes: (*a*) survey of Lu *et al.* (2008 ▸); (*b*) Bart *et al.* (2006 ▸); (*c*) bond lengths confirmed using *ab *initio** studies.

**Table 3:** Hydrogen-bond geometry (Å, °)

*D*—H⋯*A*	*D*—H	H⋯*A*	*D*⋯*A*	*D*—H⋯*A*
C4—H41⋯Cl25^i^	0.95	2.75	3.666 (3)	162

Symmetry code: (i) 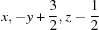.

**Table 4:** Experimental details

Crystal data	
Chemical formula	[ZnCl_2_(C_20_H_17_BrN_2_)]·0.5CH_2_Cl_2_
*M*_r_	544.02
Crystal system, space group	Monoclinic, *P*2_1_/*c*
Temperature (K)	110
*a*, *b*, *c* (Å)	13.7338 (3), 11.25476 (16), 15.2274 (3)
β (°)	114.654 (3)
*V* (Å^3^)	2139.14 (14)
*Z*	4.0
Radiation type	Mo *K*α
μ (mm^−1^)	3.40
Crystal size (mm)	0.55 × 0.40 × 0.32

Data collection	
Diffractometer	Oxford Diffraction Xcalibur (Ruby, Gemini ultra)
Absorption correction	Analytical (*CrysAlis PRO*; Oxford Diffraction, 2007 ▸)
*T*_min_, *T*_max_	0.322, 0.457
No. of measured, independent and observed [*I* > 2.0σ(*I*)] reflections	23484, 5308, 4127
*R*_int_	0.036
(sin θ/λ)_max_ (Å^−1^)	0.689

Refinement	
*R*[*F* ^2^ > 2σ(*F* ^2^)], *wR*(*F* ^2^), *S*	0.031, 0.080, 0.97
No. of reflections	5308
No. of parameters	263
No. of restraints	58
H-atom treatment	H-atom parameters not refined
Δρ_max_, Δρ_min_ (e Å^−3^)	1.67, −1.68

Computer programs: *CrysAlis PRO* (Oxford Diffraction, 2007 ▸), *SIR92* (Altomare *et al.*, 1994 ▸), *CRYSTALS* (Betteridge *et al.*, 2003 ▸), *Mercury* (Macrae *et al.*, 2006 ▸) and *ORTEP-3 for Windows* (Farrugia, 2012 ▸).

## supplementary crystallographic information

### Crystal data

**Table:**

[ZnCl_2_(C_20_H_17_BrN_2_)]0.5CH_2_Cl_2_	*F*(000) = 1084
*M**_r_* = 544.02	*D*_x_ = 1.689 Mg m^−^^3^
Monoclinic, *P*2_1_/*c*	Mo *K*α radiation, λ = 0.7107 Å
Hall symbol: -P 2ybc	Cell parameters from 12051 reflections
*a* = 13.7338 (3) Å	θ = 3.8–29.4°
*b* = 11.25476 (16) Å	µ = 3.40 mm^−^^1^
*c* = 15.2274 (3) Å	*T* = 110 K
β = 114.654 (3)°	Block, colourless
*V* = 2139.14 (14) Å^3^	0.55 × 0.40 × 0.32 mm
*Z* = 4.0	

### Data collection

**Table:**

Xcalibur, Ruby, Gemini ultra diffractometer	5308 independent reflections
Radiation source: Enhance (Mo) X-ray Source	4127 reflections with *I* > 2.0σ(*I*)
Graphite monochromator	*R*_int_ = 0.036
Detector resolution: 10.3533 pixels mm^-1^	θ_max_ = 29.3°, θ_min_ = 3.8°
ω scans	*h* = −18→17
Absorption correction: analytical (CrysAlis PRO; Oxford Diffraction, 2007)	*k* = −14→14
*T*_min_ = 0.322, *T*_max_ = 0.457	*l* = −20→20
23484 measured reflections	

### Refinement

**Table:**

Refinement on *F*^2^	Hydrogen site location: difference Fourier map
Least-squares matrix: full	H-atom parameters not refined
*R*[*F*^2^ > 2σ(*F*^2^)] = 0.031	Method = Modified Sheldrick *w* = 1/[σ^2^(*F*^2^) + ( 0.05*P*)^2^ + 0.0*P*] , where *P* = (max(*F*_o_^2^,0) + 2*F*_c_^2^)/3
*wR*(*F*^2^) = 0.080	(Δ/σ)_max_ = 0.021
*S* = 0.97	Δρ_max_ = 1.67 e Å^−^^3^
5308 reflections	Δρ_min_ = −1.68 e Å^−^^3^
263 parameters	Extinction correction: Larson (1970), Equation 22
58 restraints	Extinction coefficient: 50 (10)
Primary atom site location: structure-invariant direct methods	

### Fractional atomic coordinates and isotropic or equivalent isotropic displacement parameters (Å^2^)

**Table:**

	*x*	*y*	*z*	*U*_iso_*/*U*_eq_	Occ. (<1)
Zn1	0.24497 (2)	0.66355 (2)	0.19030 (2)	0.0137	
N2	0.21816 (15)	0.59443 (17)	0.05484 (14)	0.0135	
C3	0.20764 (19)	0.6512 (2)	−0.02501 (18)	0.0160	
C4	0.1773 (2)	0.5966 (2)	−0.11413 (18)	0.0208	
C5	0.1573 (2)	0.4766 (2)	−0.11927 (18)	0.0212	
C6	0.1680 (2)	0.4147 (2)	−0.03669 (18)	0.0174	
C7	0.19888 (18)	0.4754 (2)	0.04920 (17)	0.0131	
C8	0.22025 (19)	0.4160 (2)	0.14363 (17)	0.0125	
C9	0.20005 (19)	0.2860 (2)	0.14404 (17)	0.0142	
C10	0.0955 (2)	0.2442 (2)	0.09284 (18)	0.0183	
C11	0.0740 (2)	0.1235 (2)	0.09313 (19)	0.0213	
C12	0.1553 (2)	0.0442 (2)	0.14188 (19)	0.0223	
C13	0.2597 (2)	0.0854 (2)	0.19275 (19)	0.0209	
C14	0.2819 (2)	0.2055 (2)	0.19430 (18)	0.0168	
N15	0.25598 (15)	0.48235 (17)	0.21897 (14)	0.0123	
C16	0.29039 (19)	0.4345 (2)	0.31477 (17)	0.0129	
C17	0.2158 (2)	0.4167 (2)	0.35422 (17)	0.0149	
C18	0.2548 (2)	0.3766 (2)	0.44935 (19)	0.0194	
C19	0.3632 (2)	0.3522 (2)	0.50168 (18)	0.0224	
C20	0.4345 (2)	0.3712 (2)	0.46047 (18)	0.0211	
C21	0.40003 (19)	0.4153 (2)	0.36666 (17)	0.0161	
C22	0.4773 (2)	0.4419 (2)	0.32364 (18)	0.0209	
C23	0.0983 (2)	0.4407 (2)	0.2971 (2)	0.0217	
Cl24	0.38192 (5)	0.76686 (6)	0.28643 (5)	0.0258	
Cl25	0.08112 (5)	0.72624 (5)	0.16364 (5)	0.0192	
Br26	0.23574 (2)	0.81580 (2)	−0.013723 (19)	0.0209	
C27	0.4520 (5)	0.0542 (4)	0.4947 (5)	0.0980	0.5000
Cl28	0.5144 (3)	0.0784 (2)	0.4268 (2)	0.1155	0.5000
Cl29	0.4668 (2)	−0.0462 (2)	0.5761 (2)	0.0653	0.5000
H41	0.1690	0.6395	−0.1703	0.0212*	
H51	0.1381	0.4360	−0.1755	0.0243*	
H61	0.1547	0.3338	−0.0417	0.0201*	
H141	0.3514	0.2333	0.2278	0.0201*	
H131	0.3119	0.0332	0.2240	0.0264*	
H121	0.1414	−0.0357	0.1418	0.0274*	
H111	0.0035	0.0963	0.0592	0.0263*	
H101	0.0408	0.2963	0.0596	0.0203*	
H231	0.0610	0.4295	0.3361	0.0276*	
H232	0.0836	0.5196	0.2717	0.0276*	
H233	0.0684	0.3865	0.2432	0.0276*	
H181	0.2047	0.3684	0.4760	0.0250*	
H191	0.3882	0.3236	0.5652	0.0242*	
H201	0.5076	0.3558	0.4952	0.0231*	
H221	0.5491	0.4250	0.3692	0.0243*	
H223	0.4709	0.5244	0.3045	0.0243*	
H222	0.4612	0.3942	0.2662	0.0243*	
H271	0.3807	0.0434	0.4461	0.0989*	0.5000
H272	0.4780	0.1254	0.5360	0.0989*	0.5000

### Atomic displacement parameters (Å^2^)

**Table:**

	*U*^11^	*U*^22^	*U*^33^	*U*^12^	*U*^13^	*U*^23^
Zn1	0.01653 (16)	0.01006 (14)	0.01384 (15)	−0.00110 (11)	0.00568 (12)	−0.00071 (11)
N2	0.0140 (10)	0.0133 (10)	0.0141 (10)	0.0004 (8)	0.0066 (8)	0.0013 (8)
C3	0.0145 (12)	0.0143 (12)	0.0191 (13)	0.0003 (9)	0.0070 (10)	0.0031 (10)
C4	0.0238 (14)	0.0236 (13)	0.0143 (12)	−0.0008 (11)	0.0073 (11)	0.0049 (11)
C5	0.0285 (15)	0.0218 (13)	0.0115 (12)	−0.0015 (11)	0.0068 (11)	−0.0007 (10)
C6	0.0186 (13)	0.0163 (12)	0.0171 (13)	0.0000 (10)	0.0072 (10)	0.0002 (10)
C7	0.0121 (12)	0.0133 (11)	0.0148 (12)	0.0011 (9)	0.0064 (9)	0.0008 (9)
C8	0.0106 (11)	0.0141 (11)	0.0135 (12)	0.0015 (9)	0.0057 (9)	0.0005 (9)
C9	0.0202 (13)	0.0126 (11)	0.0124 (12)	−0.0012 (10)	0.0094 (10)	−0.0013 (9)
C10	0.0218 (13)	0.0160 (12)	0.0185 (13)	−0.0023 (10)	0.0097 (11)	−0.0019 (10)
C11	0.0249 (14)	0.0207 (13)	0.0208 (14)	−0.0071 (11)	0.0119 (11)	−0.0047 (11)
C12	0.0410 (17)	0.0109 (12)	0.0219 (14)	−0.0064 (11)	0.0201 (12)	−0.0026 (10)
C13	0.0327 (16)	0.0158 (12)	0.0168 (13)	0.0062 (11)	0.0128 (12)	0.0029 (10)
C14	0.0201 (13)	0.0171 (12)	0.0130 (12)	0.0004 (10)	0.0067 (10)	−0.0012 (10)
N15	0.0120 (10)	0.0132 (10)	0.0129 (10)	0.0007 (8)	0.0062 (8)	0.0010 (8)
C16	0.0194 (13)	0.0086 (11)	0.0115 (11)	−0.0007 (9)	0.0072 (10)	−0.0010 (9)
C17	0.0212 (13)	0.0094 (11)	0.0170 (12)	−0.0026 (9)	0.0110 (10)	−0.0027 (9)
C18	0.0293 (15)	0.0152 (12)	0.0206 (13)	−0.0055 (11)	0.0171 (12)	−0.0021 (11)
C19	0.0336 (16)	0.0184 (13)	0.0125 (12)	−0.0038 (11)	0.0069 (11)	0.0030 (10)
C20	0.0205 (14)	0.0210 (13)	0.0163 (13)	−0.0005 (11)	0.0023 (11)	0.0004 (11)
C21	0.0188 (13)	0.0144 (12)	0.0145 (12)	−0.0023 (10)	0.0064 (10)	−0.0024 (10)
C22	0.0164 (13)	0.0271 (14)	0.0187 (13)	−0.0003 (11)	0.0069 (11)	0.0009 (11)
C23	0.0193 (14)	0.0225 (14)	0.0283 (15)	−0.0021 (11)	0.0149 (11)	0.0013 (11)
Cl24	0.0251 (4)	0.0216 (3)	0.0236 (3)	−0.0094 (3)	0.0032 (3)	−0.0017 (3)
Cl25	0.0188 (3)	0.0157 (3)	0.0221 (3)	0.0015 (2)	0.0076 (3)	−0.0023 (2)
Br26	0.02633 (16)	0.01324 (14)	0.02203 (15)	−0.00216 (10)	0.00906 (12)	0.00460 (10)
C27	0.058 (3)	0.062 (3)	0.133 (3)	0.017 (2)	0.000 (2)	−0.027 (3)
Cl28	0.065 (2)	0.0416 (13)	0.160 (3)	0.0215 (12)	−0.0322 (17)	−0.0336 (16)
Cl29	0.0343 (12)	0.0717 (18)	0.0902 (17)	−0.0027 (12)	0.0261 (12)	−0.0279 (14)

### Geometric parameters (Å, º)

**Table:**

Zn1—N2	2.088 (2)	C14—H141	0.929
Zn1—N15	2.0778 (19)	N15—C16	1.438 (3)
Zn1—Cl24	2.1761 (7)	C16—C17	1.401 (3)
Zn1—Cl25	2.2281 (7)	C16—C21	1.395 (3)
N2—C3	1.327 (3)	C17—C18	1.393 (3)
N2—C7	1.361 (3)	C17—C23	1.504 (4)
C3—C4	1.386 (4)	C18—C19	1.391 (4)
C3—Br26	1.885 (2)	C18—H181	0.938
C4—C5	1.374 (4)	C19—C20	1.382 (4)
C4—H41	0.947	C19—H191	0.939
C5—C6	1.391 (3)	C20—C21	1.395 (3)
C5—H51	0.907	C20—H201	0.936
C6—C7	1.376 (3)	C21—C22	1.492 (4)
C6—H61	0.926	C22—H221	0.959
C7—C8	1.500 (3)	C22—H223	0.966
C8—C9	1.490 (3)	C22—H222	0.970
C8—N15	1.283 (3)	C23—H231	0.941
C9—C10	1.399 (4)	C23—H232	0.956
C9—C14	1.397 (3)	C23—H233	0.966
C10—C11	1.390 (4)	C27—Cl29^i^	1.849 (2)
C10—H101	0.921	C27—Cl28^i^	1.846 (2)
C11—C12	1.380 (4)	C27—C27^i^	1.753 (2)
C11—H111	0.938	C27—Cl28	1.618 (2)
C12—C13	1.394 (4)	C27—Cl29	1.626 (2)
C12—H121	0.919	C27—H271	0.958
C13—C14	1.384 (3)	C27—H272	0.989
C13—H131	0.894		
			
N2—Zn1—N15	79.01 (8)	C16—C17—C18	117.3 (2)
N2—Zn1—Cl24	127.60 (6)	C16—C17—C23	121.8 (2)
N15—Zn1—Cl24	114.61 (6)	C18—C17—C23	120.9 (2)
N2—Zn1—Cl25	100.92 (6)	C17—C18—C19	121.0 (2)
N15—Zn1—Cl25	109.18 (6)	C17—C18—H181	116.6
Cl24—Zn1—Cl25	118.50 (3)	C19—C18—H181	122.3
Zn1—N2—C3	129.22 (17)	C18—C19—C20	120.0 (2)
Zn1—N2—C7	112.23 (15)	C18—C19—H191	120.2
C3—N2—C7	118.2 (2)	C20—C19—H191	119.8
N2—C3—C4	123.8 (2)	C19—C20—C21	121.2 (2)
N2—C3—Br26	116.74 (18)	C19—C20—H201	120.7
C4—C3—Br26	119.45 (19)	C21—C20—H201	118.1
C3—C4—C5	117.6 (2)	C20—C21—C16	117.4 (2)
C3—C4—H41	122.2	C20—C21—C22	121.4 (2)
C5—C4—H41	120.2	C16—C21—C22	121.2 (2)
C4—C5—C6	119.8 (2)	C21—C22—H221	110.4
C4—C5—H51	121.5	C21—C22—H223	109.5
C6—C5—H51	118.7	H221—C22—H223	110.2
C5—C6—C7	119.0 (2)	C21—C22—H222	110.4
C5—C6—H61	118.4	H221—C22—H222	108.8
C7—C6—H61	122.6	H223—C22—H222	107.5
C6—C7—N2	121.5 (2)	C17—C23—H231	110.3
C6—C7—C8	123.3 (2)	C17—C23—H232	113.3
N2—C7—C8	115.0 (2)	H231—C23—H232	107.6
C7—C8—C9	118.7 (2)	C17—C23—H233	110.6
C7—C8—N15	116.5 (2)	H231—C23—H233	107.6
C9—C8—N15	124.8 (2)	H232—C23—H233	107.4
C8—C9—C10	118.5 (2)	Cl29^i^—C27—Cl28^i^	106.86 (4)
C8—C9—C14	122.0 (2)	Cl29^i^—C27—C27^i^	53.59 (5)
C10—C9—C14	119.4 (2)	Cl28^i^—C27—C27^i^	53.36 (5)
C9—C10—C11	119.8 (2)	Cl29^i^—C27—Cl28	13.11 (5)
C9—C10—H101	120.3	Cl28^i^—C27—Cl28	119.62 (4)
C11—C10—H101	119.9	C27^i^—C27—Cl28	66.26 (4)
C10—C11—C12	120.5 (2)	Cl29^i^—C27—Cl29	119.83 (4)
C10—C11—H111	119.3	Cl28^i^—C27—Cl29	13.31 (5)
C12—C11—H111	120.2	C27^i^—C27—Cl29	66.24 (4)
C11—C12—C13	119.9 (2)	Cl28—C27—Cl29	132.35 (5)
C11—C12—H121	120.7	Cl29^i^—C27—H271	102.3
C13—C12—H121	119.4	Cl28^i^—C27—H271	107.3
C12—C13—C14	120.1 (2)	C27^i^—C27—H271	118.0
C12—C13—H131	119.2	Cl28—C27—H271	99.8
C14—C13—H131	120.7	Cl29—C27—H271	105.3
C9—C14—C13	120.2 (2)	Cl29^i^—C27—H272	105.3
C9—C14—H141	119.3	Cl28^i^—C27—H272	108.5
C13—C14—H141	120.5	C27^i^—C27—H272	116.6
Zn1—N15—C8	114.62 (16)	Cl28—C27—H272	97.1
Zn1—N15—C16	123.06 (15)	Cl29—C27—H272	100.4
C8—N15—C16	122.0 (2)	H271—C27—H272	125.2
N15—C16—C17	119.9 (2)	C27^i^—Cl28—C27	60.38 (4)
N15—C16—C21	116.9 (2)	C27^i^—Cl29—C27	60.17 (4)
C17—C16—C21	123.0 (2)		

Symmetry code: (i) −*x*+1, −*y*, −*z*+1.

### Hydrogen-bond geometry (Å, º)

**Table:**

*D*—H···*A*	*D*—H	H···*A*	*D*···*A*	*D*—H···*A*
C4—H41···Cl25^ii^	0.95	2.75	3.666 (3)	162

Symmetry code: (ii) *x*, −*y*+3/2, *z*−1/2.
