# Supplementary figures and images for: Crystal structure of {N-[(6-bromo­pyridin-2-yl)(phen­yl)methyl­idene]-2,6-di­methyl­aniline-κ2 N,N′}di­chlorido­zinc di­chloro­methane hemisolvate
Source: Acta Crystallogr E Crystallogr Commun. 2017 Jun 2;73(Pt 7):932–5. doi: 10.1107/S2056989017007812 (PMC5499262; doi:10.1107/S2056989017007812)

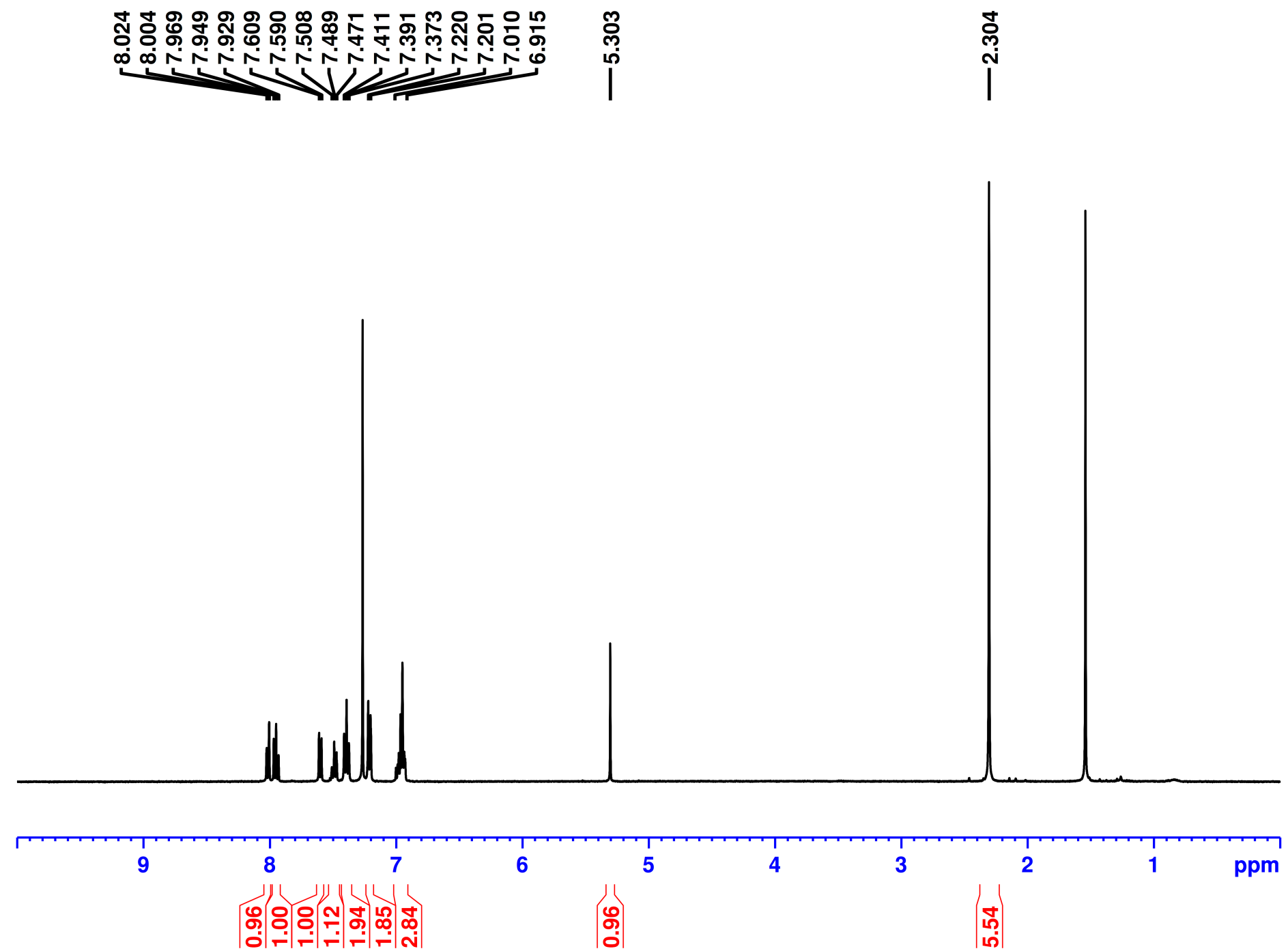

Supplement: Supplementary file 3 [file e-73-00932-sup4.pdf]

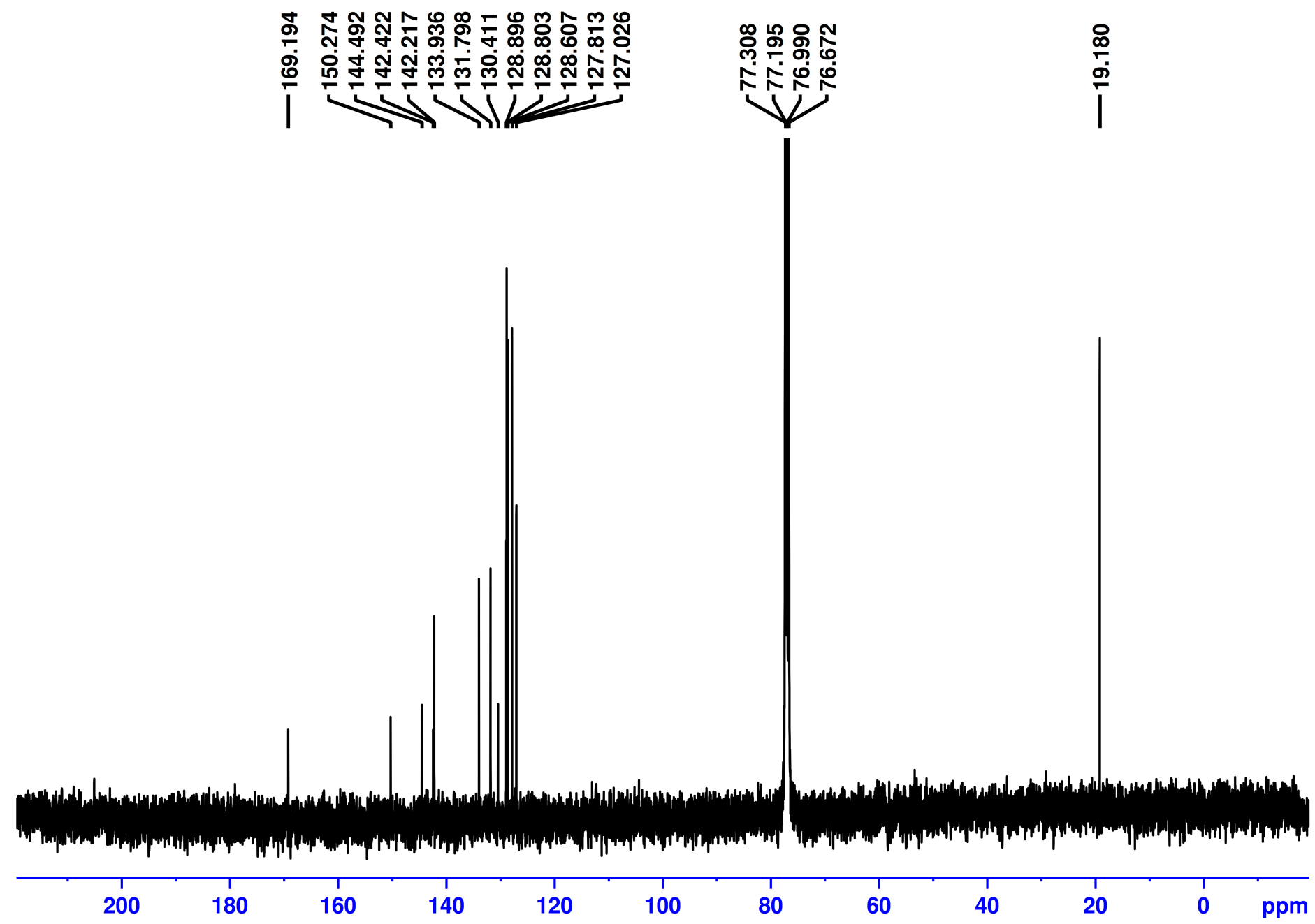

Supplement: Supplementary file 4 [file e-73-00932-sup5.pdf]
